# A system for the study of roots 3D kinematics in hydroponic culture: a study on the oscillatory features of root tip

**DOI:** 10.1186/s13007-024-01178-3

**Published:** 2024-04-01

**Authors:** Valentina Simonetti, Laura Ravazzolo, Benedetto Ruperti, Silvia Quaggiotti, Umberto Castiello

**Affiliations:** 1 Department of General Psychology, University of Padova, Padova, Italy; 2 Department of Agronomy, Food, Natural Resources, Animals and Environment (DAFNAE), University of Padova, Agripolis, Italy

**Keywords:** Root development, Hydroponics, Kinematics, Root imaging, Time-lapse, Stereovision, 3D motion analysis, Nutation, Maize

## Abstract

**Background:**

The root of a plant is a fundamental organ for the multisensory perception of the environment. Investigating root growth dynamics as a mean of their interaction with the environment is of key importance for improving knowledge in plant behaviour, plant biology and agriculture. To date, it is difficult to study roots movements from a dynamic perspective given that available technologies for root imaging focus mostly on static characterizations, lacking temporal and three-dimensional (3D) spatial information. This paper describes a new system based on time-lapse for the 3D reconstruction and analysis of roots growing in hydroponics.

**Results:**

The system is based on infrared stereo-cameras acquiring time-lapse images of the roots for 3D reconstruction. The acquisition protocol guarantees the root growth in complete dark while the upper part of the plant grows in normal light conditions. The system extracts the 3D trajectory of the root tip and a set of descriptive features in both the temporal and frequency domains. The system has been used on *Zea mays* L. (B73) during the first week of growth and shows good inter-reliability between operators with an Intra Class Correlation Coefficient (ICC) > 0.9 for all features extracted. It also showed measurement accuracy with a median difference of < 1 mm between computed and manually measured root length.

**Conclusions:**

The system and the protocol presented in this study enable accurate 3D analysis of primary root growth in hydroponics. It can serve as a valuable tool for analysing real-time root responses to environmental stimuli thus improving knowledge on the processes contributing to roots physiological and phenotypic plasticity.

## Background

Plants are able to sense environmental changes and accomplish adequate problem-solving physiological and developmental responses [1–5]. The root is the plant organ devoted to water and nutrients uptake, meanwhile providing anchorage to the plant [6]. The root apex is involved in the perception of many environmental stimuli [7], such as gravity [8, 9], light [10, 11], electric fields [12], moisture gradient [13, 14] and nitrate [15–18]. Further, it has been hypothesized that the root apex exhibits computational features related to sensory integration [2, 19].

Investigating root growth dynamics as a mean of their interaction with the environment is of key importance for improving knowledge in plant behaviour, biology and agriculture. Roots movements represent an instance of active behaviour [20] calling for an effective kinematical investigation of the circumnutating movement of primary roots, a key component of exploratory behaviour and substrate penetration [6, 21–27]. What is needed is the implementation of specific and repeatable protocols for in vivo measurements on a short time scale enabling the investigation of how plants cope with frequent and unexpected environmental changes that drive readjustments of root growth and development [7].

A variety of studies underline the importance of implementing tools for high throughput analysis of roots features [28, 29]. To date, it is difficult to study roots movements from a dynamic point of view given that available technologies for root imaging focus mostly on static characterizations, lacking temporal and three-dimensional (3D) spatial information.

Root features are usually evaluated only at the time the root system is extracted from the soil (e.g., Liu et al. [30]) or without considering repeated imaging of the roots as for tomography scans (e.g., Zeng et al. [31]). Time-lapse techniques, able to capture dynamical changes in root growth, may help to a better characterization of roots movements but, to date, they are chiefly based on two-dimensional (2D) approaches [32]. Tomography scans and Magnetic Resonance Imaging (MRI) have been used recently in studies involving repeated imaging and 3D time-lapse reconstruction: an example for tomographic scans is described in the study by Keyes et al. [33] while an example for MRI is the work by Popova et al. [34]. These technologies, however, face limitations related to imaging sampling rate which can take from few minutes to hours. Additionally, the acquisition setup typically involves the movement of the plant inside the scanner, constrained by specific dimensions and limited field of view. Despite these aspects, tomography scans and MRI remain a valuable choice for conducting in vivo measurements for appropriate experimental setups. Another method for achieving 3D time-lapse reconstruction is described in the work by Clark et al. [35]. In this study, the authors set up a system based on a custom rotating mechatronic platform that captures images of the roots from different angles enabling in this way 3D reconstruction. However, the system may result difficult to use and to adapt to different experimental configurations. Light-sheet microscopy for root imaging [36] has not been considered here because of its small field of view (from tens of micrometres to few millimetres) but it might be a valuable choice to achieve 3D imaging of root when a smaller field of view is acceptable.

In the present paper, we describe a fairly simple and affordable system with high time resolution that enables the 3D reconstruction and analysis of roots growing in hydroponics. Hydroponics is frequently used in studies requiring the control of nutrients and allow accessibility to the root system, since it is appropriate for cultivation of the plants throughout their entire life cycle and allows to perform independent experiments in reproducible root-environment conditions [37].

## Materials and methods

### Experimental set up

**Fig. 1:**
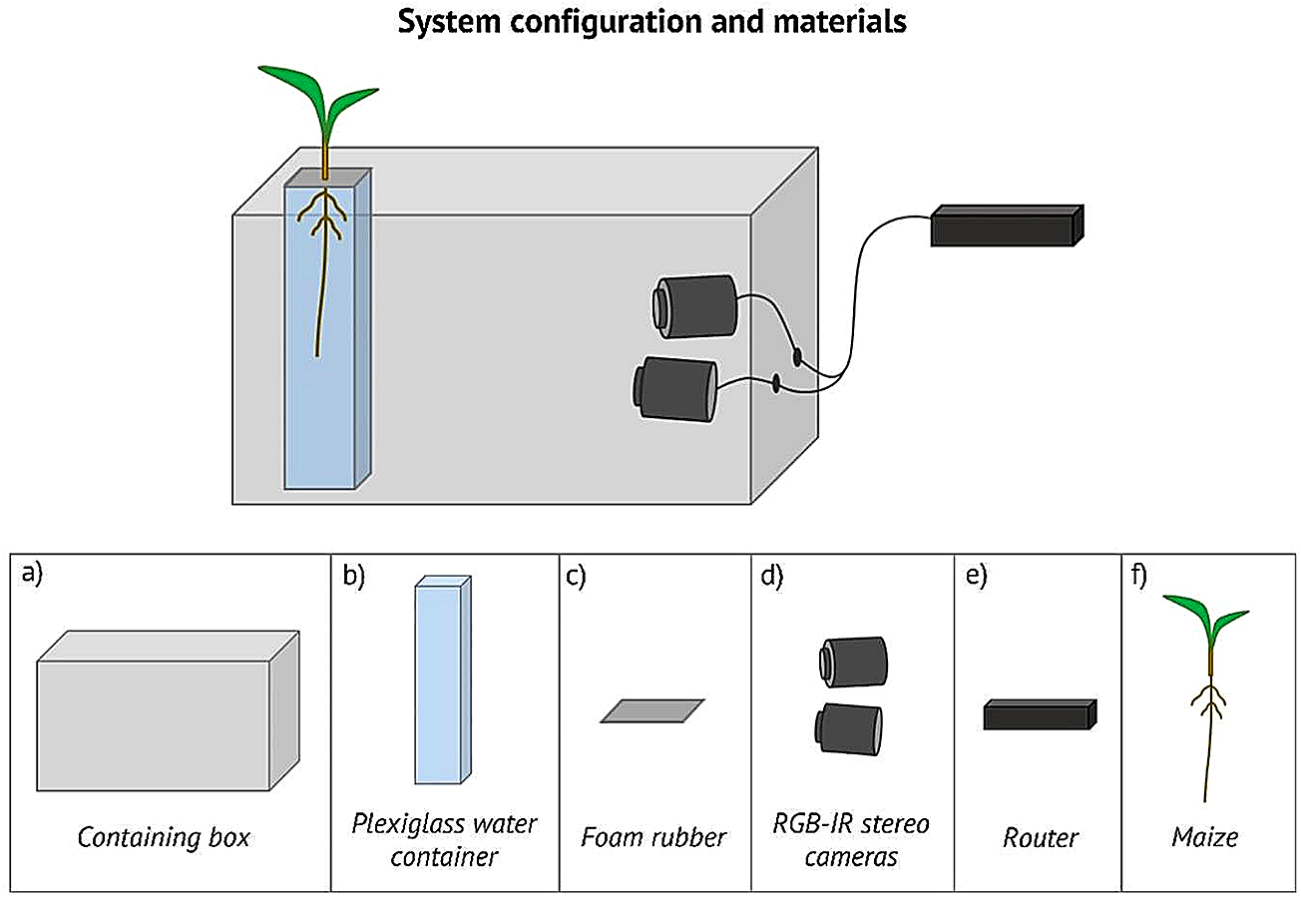
System configuration and materials. Top panel: system configuration allowing for root imaging acquisition in complete dark for the entire time of the experiment. The root grows in a transparent acrylic glass container filled with nutrient solution while a pair of stereo cameras take a picture every 3 min. Both the acrylic glass container and the stereo cameras are placed inside a box that guarantees that roots grow in a complete dark condition. The whole system is placed inside a growing chamber in controlled condition of light with a day/night cycle of 14/10. Bottom panel: components of the system. (**a**) Containing box: cardboard box 60cmX40cmX40cm. (**b**) Acrylic glass water container (PLEXX, Piazzola sul Brenta PD): acrylic glass container 10cmX10cmX30cm filled with nutrient solution (modified Hoagland nutrient solution [39], all chemicals were purchased from Sigma Chemicals (Sigma, St Louis, MO, USA) unless otherwise stated). (**c**) Foam rubber: punched foam rubber rectangle (11cmX11cmX1,5 cm) used to host and stabilize the seed. (**d**) RGB-IR stereo cameras: IP 2.1 Mpx outdoor varifocal IR 1080 P. Each camera is wired with Ethernet cables to a router. (**e**) Router: D-link Dsr-250n connected via Wi-Fi to a PC. (**f**) Maize: Zea mays L. (B73)

We developed a system enabling effective dynamic 3D imaging of maize root tip in hydroponic culture, which could be adapted to any configuration using transparent substrates (e.g. agar). The system is based on a pair of infrared stereo-cameras (RGB-IR stereo cameras: IP 2.1 Mpx outdoor varifocal IR 1080 P.) to acquire time-lapse images of the roots [38]. Acquired stereo images are then used for 3D reconstruction compensating for water distortion. The cameras are kept inside a box to maintain the roots in complete dark while the upper part of the plant grows in normal light conditions. The system has been tested on 19 *Zea mays* L. (B73) seedlings. Seeds were germinated on filter paper with the same seed orientation with the extremity where the root emerges facing down and they were kept in the dark at a constant temperature of 25 °C. 4-days-old seedlings were measured positioning the seedling on a horizontal surface and measuring the root from the lower tip of the seed to the root tip. Seedlings with a root length of approx. 3–4 cm were selected and placed in an acrylic glass container filled with a nutrient solution (modified Hoagland nutrient solution [39]) with a day/night cycle of 14/10 h. To stabilize the seedling, the seed was placed in a 1 cm thick foam rubber support with the root inside the solution and the upper part outside. To maximize the gas exchange with the atmosphere, four symmetrical holes of 7 mm diameter have been made in the foam rubber support. Measurements started right after the placement. Images for each plant were acquired for 7 days. The primary root of each plant was manually measured at the beginning and at the end of the acquisition using a measurement tape and placing the plant on a horizontal surface. Figure 1 shows the system configuration and the materials used.

### Protocol for calibration, images acquisition and 3D trajectory extraction

The protocol implemented to obtain 3D root tip trajectories from time-lapses of the stereo-cameras consisted of the following steps:

1. **System calibration** - Each camera was calibrated to estimate its intrinsic and extrinsic parameters. This process was performed only once for each camera and the calibration pattern used was a 10 × 7 chessboard with square size of 8 mm printed on a Forex® plate. The second step was the stereo-cameras system calibration to extract relative position between the two cameras. This step was performed only once (because the relative position between the two cameras does not change in the design setup) using the same calibration pattern. The stereo-cameras system must also handle the distortion introduced by water due to refraction and the impossibility of using the pinhole camera model for calibration. We used starting assumptions as in Dolerait et al. [40] assuming that, in stereovision calibrated systems acquiring underwater objects, the virtual object point is always located above the real object point on the perpendicular of the flat refractive interface, meaning that the distortion effect is on the z (depth) axis. We implemented a water calibration process that used the same chessboard calibration pattern used for the previous calibration steps. The calibration pattern was positioned in the water container rotated around the vertical axis, tilted around 30° on the z axis and it was acquired by the stereo-cameras system. Reconstructed calibration pattern showed the effect of water distortion on the z axis. Computing the difference between the expected size of the calibration pattern and its reconstructed size on the z axis we extracted the linear approximation of water distortion. Applying the resulting linear function for water distortion compensation to the reconstructed points we obtained corrected 3D points coordinates.
2. **Time-lapse acquisition** – Each camera acquired an image with a time interval of 3 min. Studies adopting a similar approach on root imaging report a chosen time interval between 2 and 4 min [32, 41]. Popova et al. [41] reports circumnutation periods for maize root tip that range between 30 and 120 min, so our choice for a 3 min interval between pictures guarantees that these movements are captured. The system supports also lower time intervals (up to few seconds) that may be used for other experiments exploring faster root tip movements. A C + + script handled the images acquisition from the cameras: it implemented a time counter set to the specified time interval and, when the counter reached the threshold value, it accessed the IP of the camera which was connected to the local network through a router (using the same approach as in Simonetti et al. [38]). This process guaranteed the synchronization of the two cameras acquisitions. Possible time shift between cameras acquisition due to network connection and threads execution are less than 1 s.
3. **Video processing** – Acquired time-lapses were processed using SPROUTS software (Ab.Acus srl Milan, Italy; [35]). The software is a standalone desktop application that allows to select and perform semi-automatic tracking of specific key points in the video. The outcome of this step is the extraction of 2D trajectories from the time-lapses of both the left and the right camera.
4. **3D trajectory extraction** – 3D trajectory of the selected points was extracted triangulating the 2D trajectories extracted from the left and right camera. The process was implemented in a Python script using OpenCV library. First, the script computed the projection matrices by multiplying the camera matrix by the rotation and translation matrix obtained during the calibration step. The projection matrices were then used to triangulate the 2D trajectories implementing a direct linear transformation. Finally, the 3D points obtained were compensated for water distortion using the linear function for water distortion compensation extracted in step 1.

**Fig. 2:**
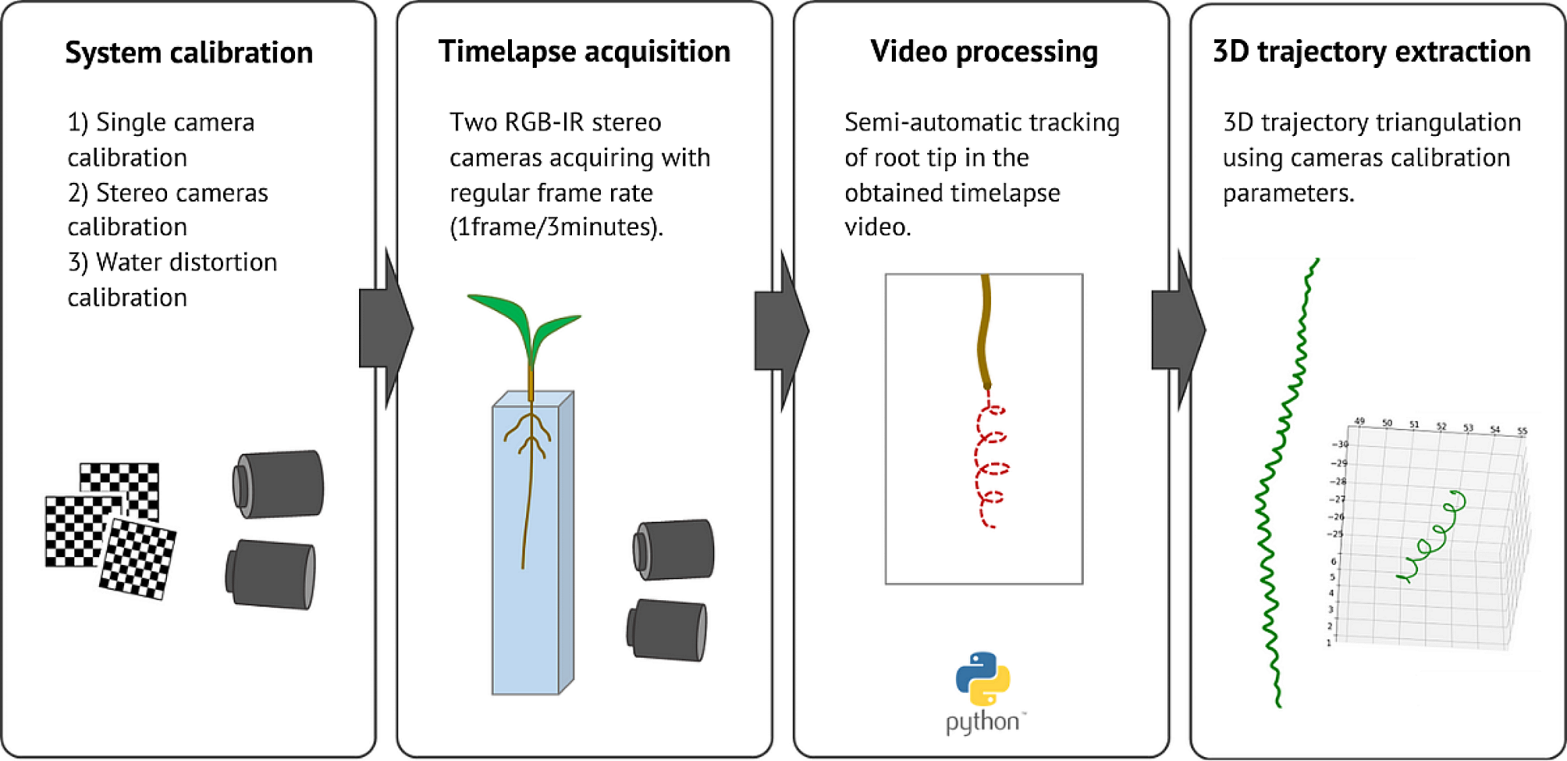
Acquisition and processing protocols used to extract the 3D trajectory of root growth for each plant. The first step of system calibration is performed only once and consists of a standard single camera calibration for lens distortion using a chessboard pattern followed by stereo cameras calibration for extrinsic parameters extraction (to enable stereo vision 3D reconstruction). The system calibration includes the calibration for the distortion introduced by water. Water distortion calibration is performed using the chessboard pattern to model (linear modelling) the distortion introduced in the depth component. The time-lapse acquisition step consists of a simultaneous acquisition of pictures by the two cameras every 3 min. The obtained time-lapse video is processed in the video processing step using a semi-automatic tracking software [38]. The 2D trajectories obtained by each camera are then triangulated using the calibration parameters to extract the 3D trajectory of the root growth

**Fig. 3:**
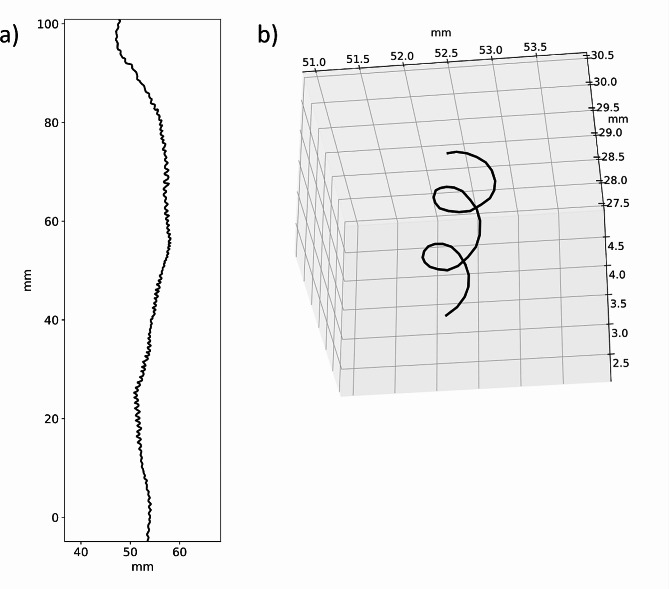
Outcomes of the root tip 3D trajectory reconstruction. (**a**) Example of trajectory reconstruction for 6 days acquisition on the xy plane. (**b**) Zoom on part of the 3D trajectory of the same root as in point (**a**) focusing on the circumnutation movement reconstruction

All the protocol steps are summarized in Fig. 2. Figure 3 shows examples of 3D trajectory reconstruction.

### Features extraction

The system extracts the 3D trajectory of the root tip and a set of descriptive features.

Extracted features are 3D versions of 2D features found in available literature [25, 41–43]:

#### Preliminary processing – root growth main component extraction

**Fig. 4:**
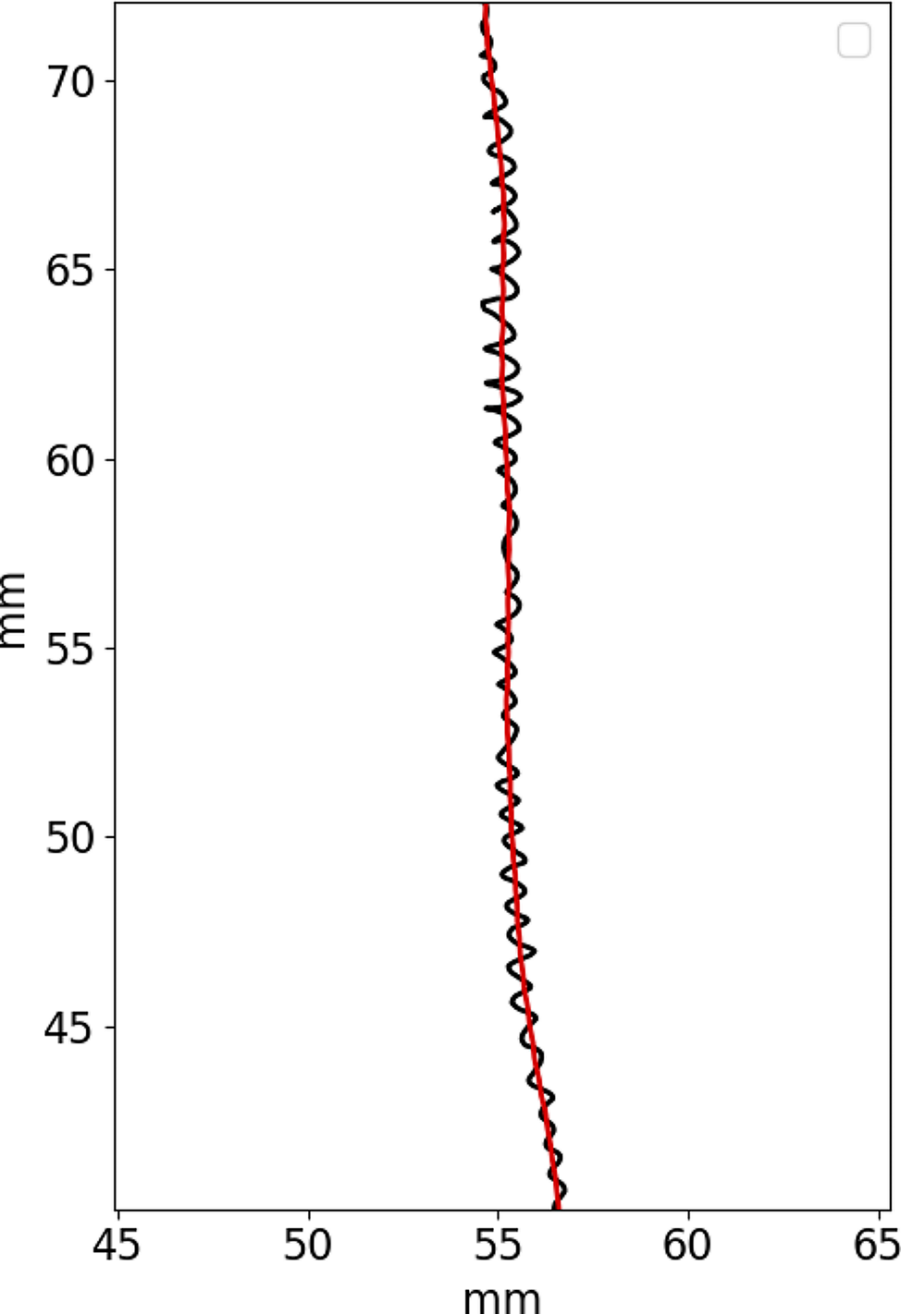
Example of the root main component of growth. Root trajectory as extracted from the triangulation step are represented in black while the root main component of growth is represented in red

3D root tip trajectory was pre-processed to enable the calculation of other features. The pre-processing step computes the main component of root growth to correctly handle and differentiate it from the oscillatory movements performed by the root tip. The main component of root growth was computed performing a moving average on the 3D trajectory coordinates with a centered window of N samples, where N was equal to the number of samples acquired in 4 h. The 4 h time window should guarantee that the root main component of growth extracted is not affected by the circumnutation movements (considering circumnutation periods in the range 30–120 min as reported by Popova et al. [41] while taking into accont the drifts of the root that are slower than 120 min). In our case, with an acquisition period of 3 min, we applied a 80-samples moving average. An example of the extraction of the root main component of growth is shown in Fig. 4 where the root tip trajectory extracted from the triangulation process is plotted in black while the root main component of growth is plotted in red. In the figure, the oscillatory movements of the root tip are clearly visible.

#### Overall primary root length

The overall primary root length was computed as the discrete sum of the 3D euclidean distances between samples of the root main component of growth:

1 $$\begin{aligned}& Overall\ primary\ root\ length\\&\quad\quad= \sum _{\varvec{i}=1}^{\varvec{N}}\varvec{E}\varvec{u}\varvec{c}\varvec{l}\varvec{i}\varvec{d}\varvec{e}\varvec{a}\varvec{n} \varvec{d}\varvec{i}\varvec{s}\varvec{t}\varvec{a}\varvec{n}\varvec{c}\varvec{e} \left(\varvec{p}\left(\varvec{i}\right),\varvec{p}(\varvec{i}+1)\right)\end{aligned}$$

Where:

N = number of samples along the root main component of growth.

p = sample of the root main component of growth.

#### Primary root growth rate

Root growth rate is computed both as absolute and relative root growth rate. Absolute growth rate is calculated as the discrete sum of the 3D euclidean distances between samples of the root main component of growth in a specified time interval. Relative growth rate is calculated as the discrete sum of the 3D euclidean distances between samples of the root main component of growth in a specified time interval divided by the root main component of growth length at the end of the specified time interval.

2 $$\begin{aligned}& Absolute\ primary\ root\ growth\ rate\\&\quad\quad= \sum _{\varvec{i}=\varvec{a}}^{\varvec{a}+\varvec{d}}\varvec{E}\varvec{u}\varvec{c}\varvec{l}\varvec{i}\varvec{d}\varvec{e}\varvec{a}\varvec{n} \varvec{d}\varvec{i}\varvec{s}\varvec{t}\varvec{a}\varvec{n}\varvec{c}\varvec{e} \left(\varvec{p}\left(\varvec{i}\right),\varvec{p}(\varvec{i}+1)\right)\end{aligned}$$

3 $$\begin{aligned}& Relative\ primary\ root\ growth\ rate\\&\quad\quad= \frac{\sum _{\varvec{i}=\varvec{a}}^{\varvec{a}+\varvec{d}}\varvec{E}\varvec{u}\varvec{c}\varvec{l}\varvec{i}\varvec{d}\varvec{e}\varvec{a}\varvec{n} \varvec{d}\varvec{i}\varvec{s}\varvec{t}\varvec{a}\varvec{n}\varvec{c}\varvec{e} \left(\varvec{p}\left(\varvec{i}\right),\varvec{p}(\varvec{i}+1)\right)}{{Overall\ primary\ root\ length}_{\varvec{a}+\varvec{d}}}\end{aligned}$$

Where:

a = First sample of the selected time interval.

d = Number of samples for the selected time interval.

p = Sample of the root main component of growth.

In our case, both absolute and relative growth rate were calculated over 1-day time intervals along the whole acquisition time. In this way we obtained growth rate arrays where each element of the array represented the growth rate value for the specific day.

#### Average tip velocity

Average tip velocity was calculated as the average distance travelled by the root tip per hour. First we computed the hourly tip velocity every N minutes of the whole movement (in our case every 15 min), then we computed the average for all the values obtained.

#### Maximum nutation amplitude

To compute the maximum nutation amplitude we first computed the array of euclidean distances on the XY plane between each sample on the root tip trajectory and the root main component of growth, then the maximum nutation amplitude was calculated as the maximum value of the array.

#### Frequency spectrum and main period of nutation

**Fig. 5:**
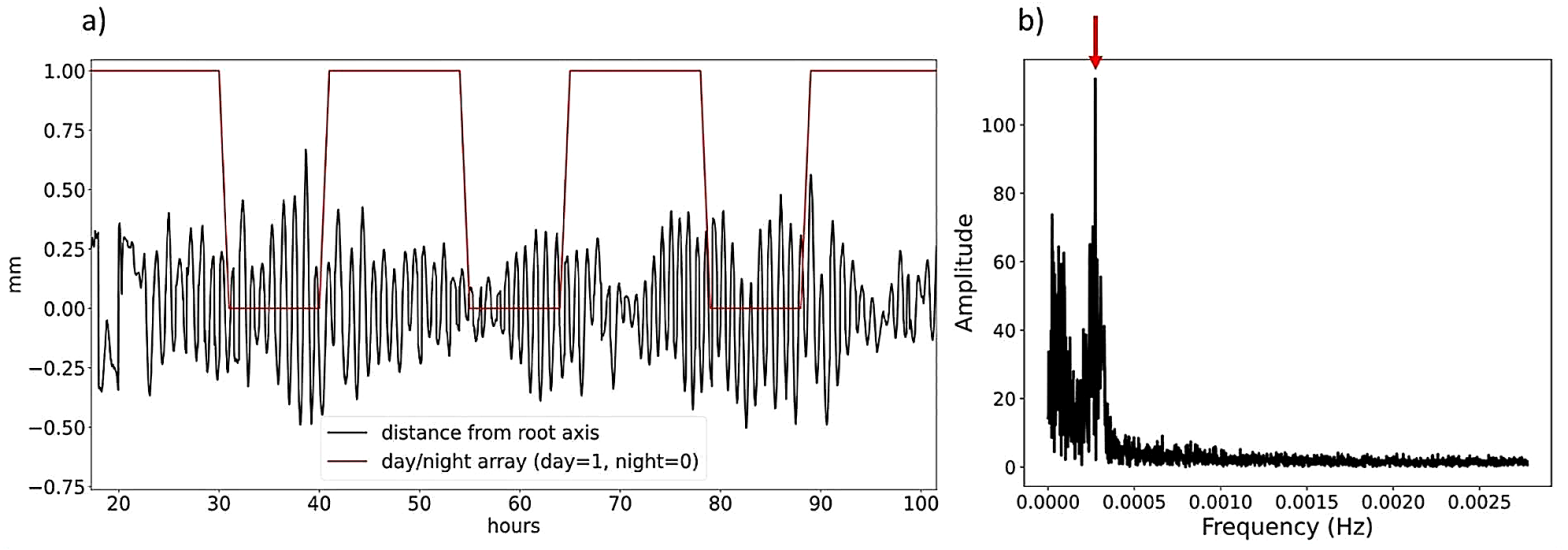
Time series of the distance of the root tip from the root main component of growth obtained computing the euclidean distance between the trajectory and the main root component of growth on the xy plane along time. Root main component of growth is computed applying a 3D moving average on the trajectory with 80 samples window. Red line represents the light/dark cycle (day = 1, night = 0). (**b**) Frequency spectrum of the signal shown in (**a**) obtained computing the one-dimensional Discrete Fast Fourier Transform using Python Numpy package. Red arrow shows the frequency with highest amplitude in the spectrum, corresponding, in this case, to a period of around 1 h

First we computed the array of euclidean distances on the XY plane between each sample on the root tip trajectory and the root main component of growth to obtain a time series like the one shown in Fig. 5a. The time series obtained was then used to calculate its Discrete Fourier Transform (DFT) by means of the Fast Fourier Transform (FFT) algoritm. The main period of nutation was computed as the period corresponding to the frequency in the power spectrum obtined with the FFT where the maximum amplitude value was detected (as shown in Fig. 5b).

## Results and discussion

The system showed good inter-reliability between operators with an Intra Class Correlation Coefficient (ICC) > 0.9 for all features extracted. ICC was computed comparing the results obtained from 2 different operators on 4 plants (see Table 1). The ICC has been computed using the absolute agreement of the two-way random effect model following [44].

**Table 1:** ICC values obtained comparing features over 4 plants obtained from the same 3D trajectory extracted by 2 different users

Feature	ICC
Overall primary root length	0.999
Absolute primary root growth rate	0.999
Relative primary root growth rate	0.997
Average tip velocity	0.991
Maximum nutation amplitude	0.963
Main period of nutation	0.999

**Fig. 6:**
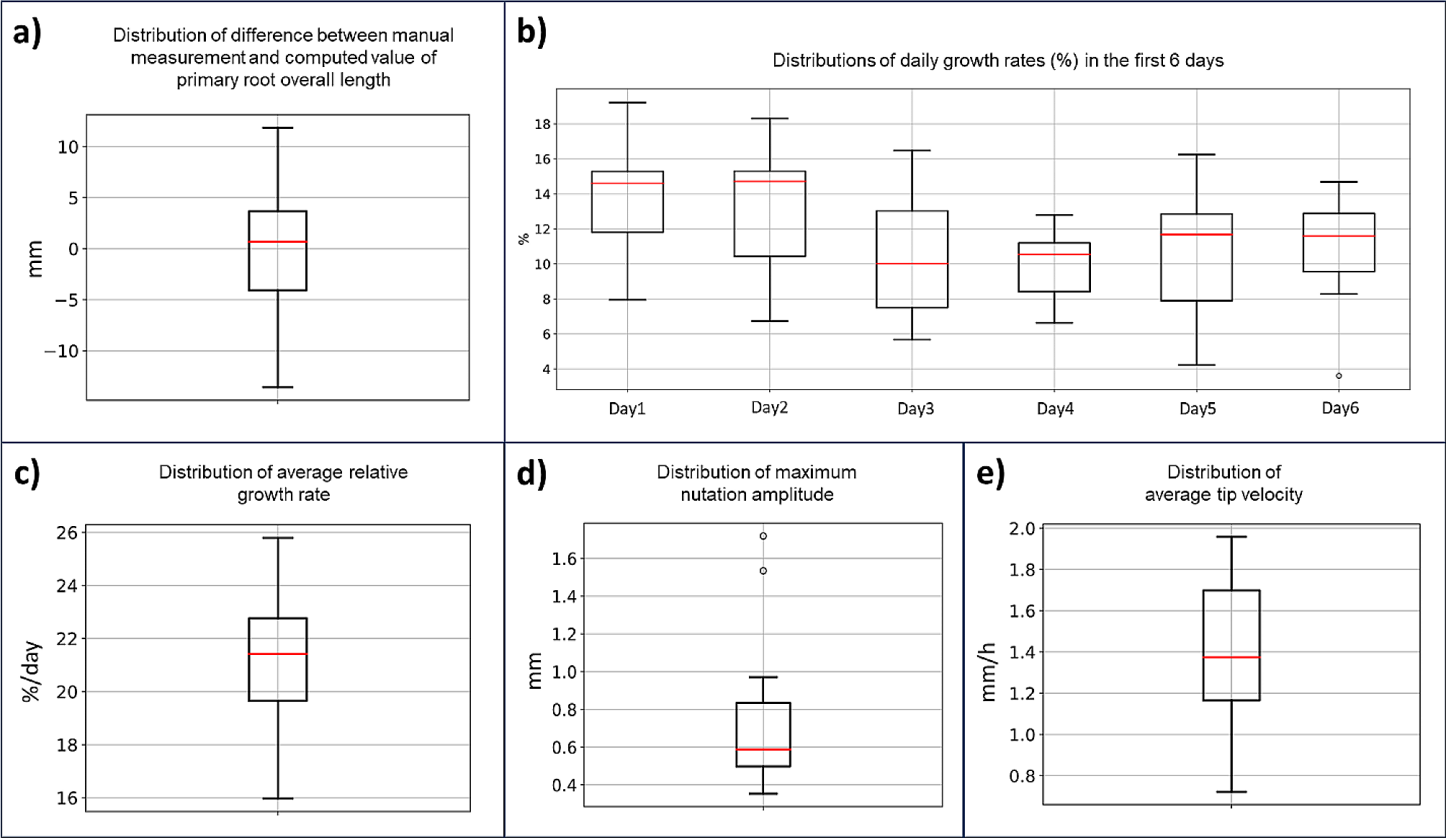
(**a**) Boxplot of the distribution of the difference (in mm) between overall root length value measured manually at the end of the experiment and overall root length value as computed by the system. Red line represents the median value. (**b**) Boxplots of the distributions of daily growth rates (%) for each of the first 6 days of the experiment. Red line represent the median values. (**c**) Boxplot of the distribution of average relative growth rate (%/day). Red line represents the median value. (**d**) Boxplot of the distribution of maximum circumnutation amplitude values computed as maximum distance from the root main component of growth. Red line represents the median value. (**e**) Boxplot of the distribution of average tip velocity (mm/h). Red line represents the median value

The system showed good measurement accuracy: comparing manual measurement of the overall primary root length acquired at the end of the experiment versus the overall primary root length computed by the system, we obtained an absolute mean difference of 0.54, an absolute median difference of 0.68 mm and a distribution of values that is below an absolute value of 5 mm within the 25th and the 75th percentile (see boxplot in Fig. 6a). The average root length measured at the end of the experiment was 20.2 cm, so the estimated mean error is 2.7% of the overall root length.

Figure 6 shows the boxplots for the distributions of the features calculated. Values obtained are in line with expected values when considering similar experiments on maize [26, 41, 43].

**Fig. 7:**
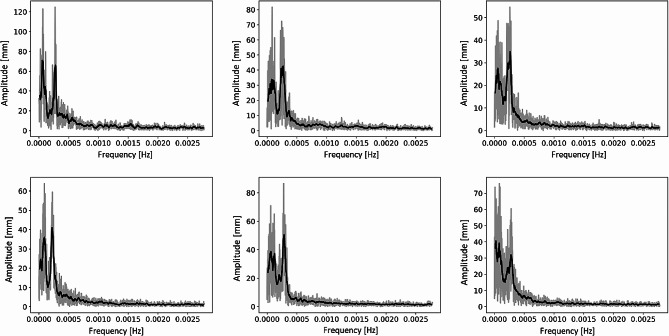
Frequency spectra for 6 sample plants. In grey, the raw data, while in black the spectrum after an 8-samples moving average

**Fig. 8:**
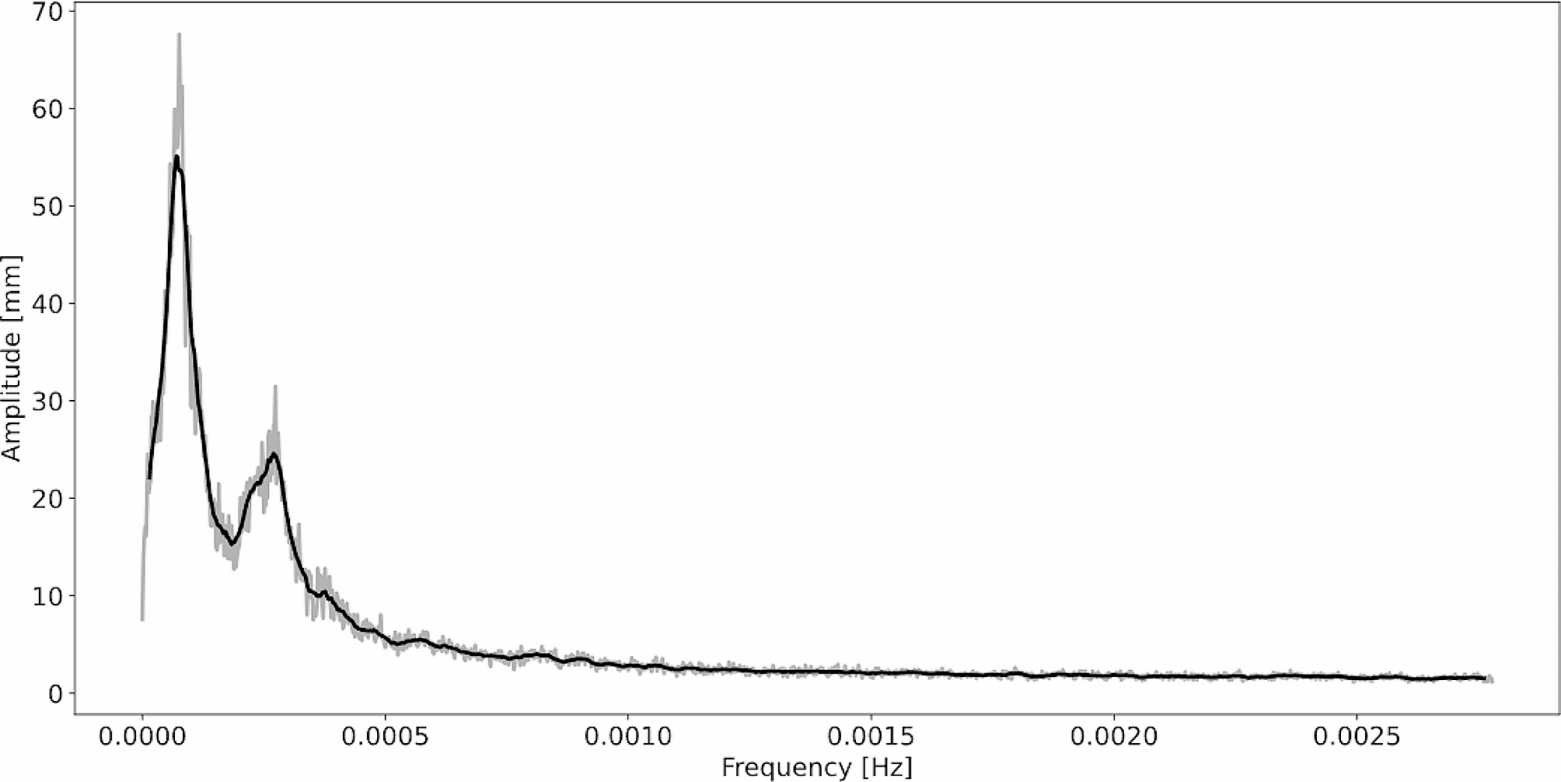
Average frequency spectrum obtained as the average of all the spectra extracted for each plant acquired. In grey, the raw data, while in black the spectrum after an 8-samples moving average

We extracted the frequency spectrum of root tip movements for all the plants as described in section “Materials and methods – Features extraction”. Analysing the frequency spectra for different plants, we identified a similar frequency amplitude distribution across plants, with two main peaks (see examples of spectra in Fig. 7). To quantitatively evaluate this trend on the whole population, we performed a frequency-based average of all power spectra extracted from all the acquired plants. For each frequency value we performed the average of the spectrum amplitude corresponding to that frequency for all the plants obtaining an average spectrum. The resulting average spectrum is shown in Fig. 8. The average spectrum clearly showed 2 peaks in the frequency amplitude. The first peak corresponded to a frequency of 0.000062 Hz and a time period of 4.5 h, while the second peak corresponded to a frequency of 0.00027 Hz and a time period of 1.02 h. The value obtained for the second peak, which was around 60 min period, is perfectly in line with the findings of Popova et al. [41] where they obtained that, in the majority of cases, circumnutations show 40–80 min periods.

## Conclusions

The system and protocol presented in this study enable accurate 3D analysis of primary root growth in hydroponic culture. Comparing the overall root length computed by the system and the manually measured root length we obtained a median difference which is less than 1 mm. The approach is easy to adopt and has the potential to become a standard for time-dependent 3D root imaging. The system has been used on maize roots to investigate their kinematic and oscillatory features during the first 7 days of growth. It resulted reliable across operators (with ICC values > 0.9 for all the features extracted as shown in Table 1) and accurate in the values obtained. Statistics extracted from the maize plants analysed showed a clear oscillatory content in the root tip movement that is the combination of two main periodic signals, one with a period of around 4 h and a second one with a period of around 1 h. In the future it would be interesting to analyse the variation of the frequency spectrum along the time of the experiment, applying, for example, a wavelet analysis approach.

Our system may become a useful tool to analyse real-time motor root responses to environmental stimuli, thus improving knowledge on processes contributing to roots physiological and phenotypic plasticity.

## Data Availability

Data describing 3D trajectories used in this paper are available here: https://zenodo.org/record/8422242. Software and scripts are available for research purposes upon request through the email address: mindtheplantlab@gmail.com.

## Abbreviations

2D: Two dimensional
3D: Three dimensional
DFT: Discrete Fourier Transform
FFT: Fast Fourier Transform
ICC: Intra Class Correlation Coefficient
